# Le nodule nécrotique solitaire du foie: à propos de deux cas

**DOI:** 10.11604/pamj.2016.25.183.10970

**Published:** 2016-11-22

**Authors:** Zeineb Mzoughi, Sana Ben Slama, Dhouha Bacha, Haifa Romdhane, Rached Bayar, Asma Sassi, Ahlem Lahmar

**Affiliations:** 1 Service de Chirurgie Viscérale, Hôpital Militaire Slim La Marsa, Tunisie; 2 Université de Tunis El Manar, Faculté de Medecine de Tunis, 1007, Tunis, Tunisie; 3 Service d’Anatomie Pathologique, Hôpital Militaire Slim La Marsa, Tunisie; 4 Service de Gastro-entérologie, Hôpital Militaire Slim La Marsa, Tunisie

**Keywords:** Nodule, solitaire, foie, Nodule, solitary, liver

## Abstract

Les lésions hépatiques nodulaires sont majoritairement tumorales. Le nodule nécrotique solitire du foie est rare. Le diagnostic preopératoire est difficile. Il s'agit d'une femme âgée de 43 ans qui était opérée d'un adénocarcinome du rectum métastatique au foie opéré. Le scanner abdominal de surveillance a révélé un nodule hépatique d'allure métastatique. Le second cas est celui d'un homme de 56 ans, admis pour cholécystectomie avec découverte per-opératoire d'un nodule hépatique. Dans les deux cas, le nodule hépatique était sous capsulaire et a été réséqué. L'examen anatomopathologique posait le diagnostic de nodule nécrotique solitaire du foie. Le nodule nécrotique non spécifique du foie est une lésion qui doit être évoquée, même dans un contexte néoplasique. Une caractérisation par l'imagerie et une ponction biopsie des lésions hépatiques peut être utile.

## Abstract (en)

Nodular liver lesions mainly consist of tumors. Solitary necrotic nodule of the liver is rare. Preoperative diagnosis is difficult. We report the case of a 43-year old woman undergoing surgery for the treatment of a rectal adenocarcinoma metastatized to the liver. Abdominal CT scan showed liver nodule mimicking liver metastasis. The second case is that of a 56 year-old man undergoing cholecystectomy in whom a liver nodule was detected intraoperatively. In both cases, liver nodule was subcapsular and was resected. Anatomopathological examination allowed the diagnosis of solitary necrotic nodule of the liver. Nonspecific necrotic nodule of the liver should be suspected in liver lesions, even in neoplastic context. Characterization using imaging studies and puncture biopsy of liver lesions can be useful.

## Introduction

Le nodule nécrotique solitaire du foie (NNS) est une lésion bénigne peu connue, décrite pour la première fois en 1983 par Shepherd et Lee [1]. Son étiopathogénie reste encore discutée. Certains auteurs suggèrent une origine infectieuse, post-traumatique ou une involution scléreuse d'un hémangiome. Le NNS est souvent de découverte fortuite et fait craindre une lésion tumorale maligne métastatique [2]. Le diagnostic histologique est de ce fait impératif. Nous rapportons deux cas de nodule nécrotique solitaire du foie.

## Patient et observation

**Figure 1:**
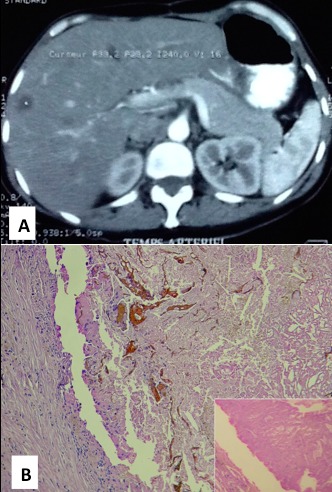
A) scanner abdominal: nodule hépatique périphérique du segment V, hypodense en cocarde, de 20x15 mm, à paroi épaisse; B) aspect histologique: lésion nécrotique avec présence de débris brunâtres, cernée d’une coque périphérique fibro-hyaline et inflammatoire avec des cellules géantes (HEx10). En cartouche, le centre du nodule correspond à un tissu de nécrose de coagulation éosinophile sans structures cellulaires ni vasculaires (HEx40)

Nous rapportons un premier cas d'une femme âgée de 43 ans, aux antécédents d'adénocarcinome du rectum opéré avec métastases hépatiques métachrones réséquées. Au cours du bilan radiologique de surveillance post-thérapeutique, le scanner abdominal a révélé un nodule hépatique périphérique du segment V de 20 mm de grand axe, évoquant une métastase hépatique nécrosée (Figure 1, A). Le second cas est celui d'un homme de 56 ans, admis pour cholécystectomie avec découverte per-opératoire d'un nodule hépatique de 8 mm de grand axe. Dans les deux cas, le nodule hépatique était sous capsulaire et a été réséqué. L'examen macroscopique était semblable avec des lésions blanc-jaunâtres, à contenu pâteux. L'examen histologique montrait un aspect comparable avec un tissu de nécrose de coagulation éosinophile, acellulaire avasculaire et entouré d'une coque fibro-hyaline (Figure 1, B). La coloration au PAS ne montrait pas de membranes hydatiques ou de microorganismes infectieux. Ces aspects étaient tout à fait compatibles avec un NNS du foie.

## Discussion

Le NNS est une lésion bénigne rare qui touche avec prédilection l'adulte de sexe masculin. L'âge de survenue varie entre 50 et 70 ans. Cette lésion est le plus souvent unique, sous capsulaire et au niveau du lobe droit du foie. Sa taille varie entre 3 et 25 mm de grand axe. Elle est le plus souvent de découverte fortuite et l'aspect radiologique peut être très utile [2]. Ainsi, cette lésion apparaît au scanner homogène hypodense et non réhaussée par le produit de contraste. L'IRM est plus évocatrice lorsque la lésion est unique, avasculaire et en hyposignal T1 et T2 [3]. Lors d'un contexte néoplasique comme notre premier cas, une métastase hépatique nécrosée ne peut être éliminée et une preuve histologique est impérative. Histologiquement, le NNS du foie correspond à un matériel nécrotique éosinophile central, avec parfois présence de cristaux de cholestérol ou de calcifications. L'absence de résorption du matériel nécrotique peut s'expliquer par la présence d'une coque fibro-hyaline. Le type de cellules présentes dans la coque ou autour du nodule pourrait expliquer les hypothèses étiologiques avancées : la présence de polynucléaires éosinophiles orienterait vers une origine parasitaire et les cellules épithélioïdes plutôt vers l'origine tuberculeuse. Néanmoins, ces hypothèses n'ont jamais été confirmées par le reste des explorations clinico-biologiques [4]. Le traitement du NNS est chirurgical. Certaines équipes préconisent une surveillance radiologique après preuve histologique par ponction biopsie [5]. Cependant, la nécrose de coagulation peut être très difficile à différencier d'une nécrose tumorale portant à tort ce diagnostic. L'exérèse totale et chirurgicale reste donc la règle.

## Conclusion

Le NNS est une lésion de diagnostic préopératoire difficile surtout dans un contexte néoplasique. Cependant, une caractérisation radiologique et une ponction biopsie peuvent être utiles pour la suspecter. La résection chirurgicale reste la règle. Le diagnostic histologique pourrait éviter un sacrifice parenchymateux hépatique.
